# Facilitating writing performance of EFL learners *via* virtual reality: Immersion, presence, embodiment

**DOI:** 10.3389/fpsyg.2023.1134242

**Published:** 2023-05-05

**Authors:** Baoxin Feng, Lee Luan Ng

**Affiliations:** Faculty of Languages and Linguistics, Universiti Malaya, Kuala Lumpur, Malaysia

**Keywords:** immersive virtual reality (IVR), EFL, writing education, embodiment experience, sense of presence

## Abstract

Immersive virtual reality (IVR) technology is being used to help EFL learners overcome their difficulties with their language skills, especially writing skills. Past studies showed that the instrument that provides immersion will positively affect learners’ written performance. In line with that, this study aims to investigate the vocabulary usage and writing performance of learners who learn vocabulary *via* IVR versus those who learn from conventional classroom-based instruction. A total of 144 Chinese-speaking English learners, who were divided into experimental group (*N* = 69) and control group (*N* = 75), experienced the treatments related to the writing tasks. The results show that the learners in the experimental group wrote more informatively and presented more details. Comparative analysis revealed that learners using IVR performed significantly better on target word usage, lexical density, distribution richness, and completion of task than those in the conventional classroom. Based on the results, it would seem that the positive transfer of learning may be related to the experience of exploring in a virtual environment. The immersion of IVR and the sense of presence and embodiment enable learners to benefit from their immersive experience which aids the use of vocabulary in their writing. The implication of the study demonstrated the impact of the technological factors, whereby what causes the improvement in writing performance is due to the learners’ virtual experience and their sense of embodiment.

## 1. Introduction

By simulating an interactive scene, virtual reality (VR) technology makes users believe they are instantaneously in another realm, which may bring in more possibilities for language teaching and learning. Learners may explore and learn in a less space-constraining and more interactive environment without the usual physical limiting attributes of a traditional classroom. Based on the Scopes database, there have been over 1,200 studies related to VR-based education in recent years (Makransky and Petersen, 2021) and the number is increasing. This phenomenon is evident based on the recent published studies on how VR technology promotes L2 learning (Lan et al., 2019; Alfadil, 2020; Tai et al., 2020; Lai and Chen, 2021). According to Qiu et al. (2021), studies in this area have gone through the *technology exploration stage* (2008 to 2013), the *desktop VR development stage* (2014 to 2016), and the *immersive VR*/*AR promotion stage* (2017 to 2019). The empirical studies that used different stages of VR technology provided their participants with different learning experiences in virtual environments. For example, learners using desktop-based VR could only control the movement of the on-screen avatar by using the mouse and keyboard, which come across as more of a third-person perspective in terms of how the individual learners experience the process. In recent years, when VR has undergone a transition from a computer-based application to more stand-alone wearable devices, they enabled individuals to feel as if they have been teleported into the particular environment and experienced physical-like participation. With the popularity of Helmet-Mounted Displays (HMDs), immersive virtual reality (IVR) technology is gradually coming to the forefront of researchers’ minds. IVR technology is capable of replacing the user’s sight and capture their body movements and greatly enhances the user’s immersion, which is defined as an objective measure of the vividness offered by a system, and the extent to which the system is capable of shutting out the outside world (Cummings and Bailenson, 2016). Immersion is also considered one of the unique technological factors of IVR, making it unique from other multimedia technologies.

The bulk of studies in the field of VR-assisted Language Learning focus on vocabulary learning, with relatively few studies focusing on writing (Klimova, 2021). There is consensus that IVR technology has a positive impact on vocabulary retention (Ebert et al., 2016; Yossatorn and Nimnual, 2019; Alemi and Khatoony, 2020; Alfadil, 2020). According to these empirical studies, learners using IVR technology were found performed significantly better on target vocabulary retention than learners using conventional methods, such as word cards and textbooks. This may be related to the high level of engagement and focus, as the IVR technology is able to isolate the user from real-world distractions (Huang et al., 2019). Another researcher perceives that the virtual environment helps learners to encode their knowledge based on space, which facilitates their efficiency in retrieving vocabulary (Cho, 2018). Despite the fact that better vocabulary retention is part of vocabulary instruction objectives, its fundamental purpose is to enable learners to use their new words appropriately in the real world. The ability of learners to use newly learned vocabulary actively and appropriately is an important indicator of successful vocabulary learning. To assess this competence, one method is to have students utilize the vocabulary to complete a writing task. It is critical for study in this field to investigate the contrasts in writing performance between learners utilizing IVR technology and those in conventional classrooms. It is possible to deduce how learners transfer vocabulary knowledge and apply them to achieve a certain goal from their writing. This helps to understand how learning experiences in a virtual environment affect EFL learners’ writing performance.

### 1.1. VR-assisted writing instruction

As a form of language output, writing is an approach to identify the gap between what learners want to and are able to write, so as to demonstrate their overall language proficiency in an integrated way (Lou et al., 2010). Therefore, enhancing learners’ writing performance is a crucial issue (Lan et al., 2015, 2019). With the increasing popularity of technology in the teaching of writing, IVR technology is being considered as one of the appropriate aids (Lan et al., 2016). Previous studies suggest that IVR technology may influence writing performance by promoting learners’ motivation or self-efficacy, whilst the IVR experience related to the topic can be beneficial in terms of its contribution to the content (Huang et al., 2019; Lan et al., 2019). Lan et al. (2019) designed a comparative study for investigating the differences in prewriting plans, writing performance, and learning performance among 60 English-speaking Chinese learners. Learners in the experimental group received instruction through IVR technology in scenarios related to the writing topic, while learners in the control group received instruction on the same content and procedures in a conventional classroom. The results showed that the learners in the experimental group wrote significantly longer and achieved better performance in terms of *content*, *organization*, *vocabulary,* and *grammar*. Virtual scenarios liberate students’ minds and imagination, which is thought to motivate learners and ultimately lead to improved writing performance (Lan et al., 2019). The findings of this study showed that virtual scenarios affect writing performance in L2 as well as the learning behaviors of learners. However, due to the lack of pre-test results, the researchers did not carry out a within-group comparison of performance. Whilst another empirical study investigated the impact of teaching writing based on Google Earth on the expository writing of 22 EFL learners (Chen et al., 2020). Google Earth is an application that generates a 3D panoramic view of the target location, giving the learners the experience of being there. The results showed a significant improvement in the students’ expository writing skills, particularly in the areas of *description*, *cause*, *comparison*, and *enumeration*. The study reveals that improved writing performance may be related to spatial ability, which is defined as the ability to form well-constructed visual shapes that the learners formed mentally (Yurt and Tünkler, 2016). The limitation of the study being the sample size of the study was small, which affected the reliability of the statistical analysis. In addition, the involvement of a control group may help to understand whether the improvement in expository writing is more significant with IVR technology compared to the conventional classroom.

However, the findings of other studies seemed to indicate otherwise. In the study by Dolgunsöz et al. (2018), 24 EFL learners were divided into two groups who learned through a mobile VR device and a 2D video, respectively. As a result, there was no significant difference in the writing performance of the two groups of learners on a writing task. However, the limitation of the study being there is a lack of information regarding the rubrics used for scoring. Similarly, Huang et al. (2019) designed a comparative experiment based on Spherical video-based virtual reality (SVVR) technology to investigate the differences in writing performance between learners who used SVVR and those in the control group. The study revealed significant differences in the performance of the two groups of learners in terms of writing content and appearance, but not in terms of organization and vocabulary use. The limitation of the study was the use of low-immersion VR devices: smartphones were used as displays for the VR glasses; the virtual scenes were static 360° panoramas; and the teaching content was presented on 2D panels in the virtual environment. These factors may have reduced the immersion of the learners. In short, previous studies have had arguments about whether IVR enhances learners’ writing performance in terms of content, organization, vocabulary, and grammar. Based on the details of these studies, it is evident that the sample sizes were small, and there is a lack of within-group and between-group comparisons of performances between the treatment and control groups. Thus, the present study aims to address these research gaps.

### 1.2. The cognitive affective model of immersive learning

The Cognitive Affective Model of Immersive Learning (CAMIL) is a theoretical framework referred to by past research when explaining how IVR technology facilitates learning performance. According to CAMIL, there are a total of 22 pathways formed among the *technological factors*, *affective and cognitive factors*, and *learning outcomes* (please see Makransky and Petersen, 2021 for an extensive description). In line with that, immersion and interactivity are the features of IVR technology that create users’ sense of presence and agency. Presence is defined as a perception of “being there,” while agency refers to a feeling of controlling their actions in the virtual environment (Riva et al., 2003; Moore and Fletcher, 2012). This is linked to the users believing that their bodies are located in another space. Based on previous empirical studies, Makransky and Petersen (2021) believed that the affective and cognitive factors would undergo changes, and that includes embodiment. The term “embodiment” can be used to describe the experience of having a virtual body, and the possibility to feel the sensorial events directed to the body (Kilteni et al., 2012). These factors were expected to influence the outcomes of learning and knowledge transfer. Similarly, the context of the present study is to explore how IVR technology enhance learners’ learning outcomes. Thus, CAMIL theory is considered to provide a perspective for understanding the link between technological factors and learning performance.

**Figure 1:**
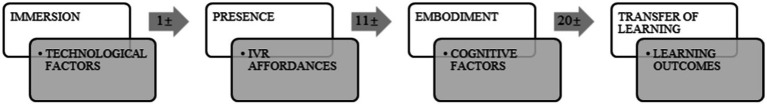
Relevant factors and pathways based on CAMIL theory.

As one of the technological factors (shown in Figure 1), immersion enables learners to instigate a sense of presence in virtual environments (path 1). This mental process is accompanied by a displacement of the learner’s self-perception (Riva et al., 2003). Based on this, these learners will be conscious about the context and believe they are part of the 3D world. For instances, when learners get used to the freedom to move around and interact with objects in the virtual environment, this triggers an embodiment experience (path 11). A series of actions are required when learners acquire the knowledge of a vocabulary when they enter a virtual room, then approach one of the objects and interact to gain information about it. Subsequently, they may go on to explore the object next to it or leave. The whole process forms an event that is accompanied by a coherent memory of that event, which may ultimately facilitate language learning and retention. In short, the embodiment aspect will contribute to the transfer of learning, which will positively impact the outcome of learning (path 20).

### 1.3. Research questions and hypotheses

This study aims to explore whether VR-assisted learning improves task-based writing in the perspectives of target vocabulary usage and writing performance. The research questions in line with the objectives are shown as follow:

*RQ1*: To what extent does the learners’ vocabulary usage performance differ between students who experience VR and conventional classroom instruction in terms of 1) *Target word usage*, 2) *Lexical density*, 3) *Distribution richness*, 4) *Spelling mistake*?

*RQ2*: What are the differences in writing performance between students who experience VR and conventional classroom instruction in terms of 5) *Completion of task*?

Consequently, a total of five hypotheses are proposed and displayed in Table 1.

**Table 1:** Overview of hypotheses 1 through 5.

*Hypothesis 1*	Learners using VR have significantly higher target vocabulary usage than learners in conventional classroom instruction.
*Hypothesis 2*	Learners using VR have significantly higher lexical density than learners in conventional classroom instruction.
*Hypothesis 3*	Learners using VR have significantly higher lexical variation than learners in conventional classroom instruction.
*Hypothesis 4*	Learners using VR have significantly higher vocabulary spelling mistakes than learners in conventional classroom instruction.
*Hypothesis 5*	Learners using VR have significantly better task completion than learners in conventional classroom instruction.

## 2. Method

### 2.1. Research design

**Figure 2:**
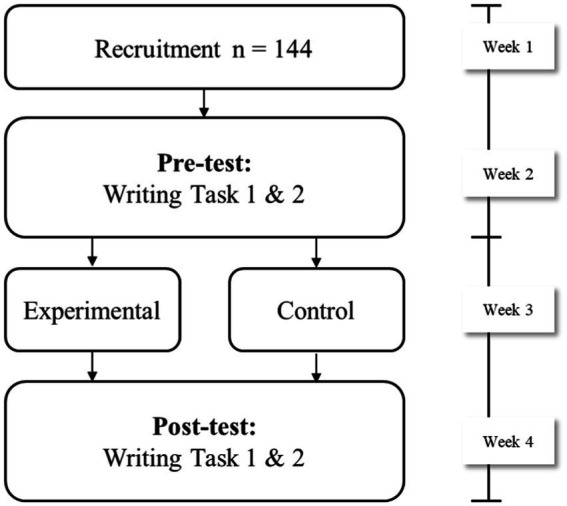
Research design.

A two-group quasi-experimental design was employed in this study (shown in Figure 2). During the recruitment phase, a brief session introducing the purpose of the experiment and the process was conducted first (week 1). Subsequently, a pre-test which lasts 60 min, was conducted. The following week, two writing tasks were carried out with the participants (week 2). Based on their performance in the pre-test, the participants with similar average scores were divided into experimental and control groups. The experimental group was given of VR-based instruction on vocabulary items that are related to the topics of both writing tasks. Whilst the control group learned the same target words under the guidance of the instructors. Learners in the experimental and control groups received the intervention for five consecutive days, with each session lasting 40 min, 200 min in total (week 3). During the sessions, due to the flexibility that allowed the learners to explore the VR environment, those in the experimental group get to be exposed to the new lexical words as they explored the virtual environment, while those in the control group learn the words in lessons that have been pre-planned by the teacher. The control group learnt an average of 20 words per session with a short review at the end of each session. A post-test consisting the same writing tasks were then administered immediately after the end of the intervention (week 4). Mixed Repeated Measures ANOVA were used to compare within-group differences between the pre-test and post-test, and between-group variability between the two groups of participants on multiple dimensions.

### 2.2. Participants

A total of 144 Chinese-speaking undergraduates were invited as the participants (Mage = 18.7, SD = 0.85), of which 129 females and 15 males. All of them are from a normal university in the middle of China. These participants had English formal education for more than 6 years. According to the average scores of the pre-test writing tasks, they were divided into experimental group (*N* = 69; *M* = 34.17, SD = 11.05) and control group (*N* = 75; *M* = 34.20, SD = 15.47). Grouping was based on the matched pairs randomization, where learners with similar scores on the pre-test were separated into two groups. During the post-test, 19 participants quite from task 2 due to the personal reason. As a result, the effective sample in task 1 is 144, whilst it is 125 in task 2.

### 2.3. Procedure

Participants were invited to a venue with a maximum capacity of 150 people. At the start of the pre-test, each participant was given a paper answer card. Task 1 was a descriptive-based writing task, whereby they have to describe and summarize information in pictures provided by the researcher; task 2 was a creative-based writing task, in which participants were asked to expound their ideas and plans for a specific topic. Task 1 and 2 were allocated 20 and 40 min, respectively. After the pre-test, the writing data was initially scored, based on which the participants were divided into experimental and control groups.

#### 2.3.1. Experimental group session

**Figure 3:**
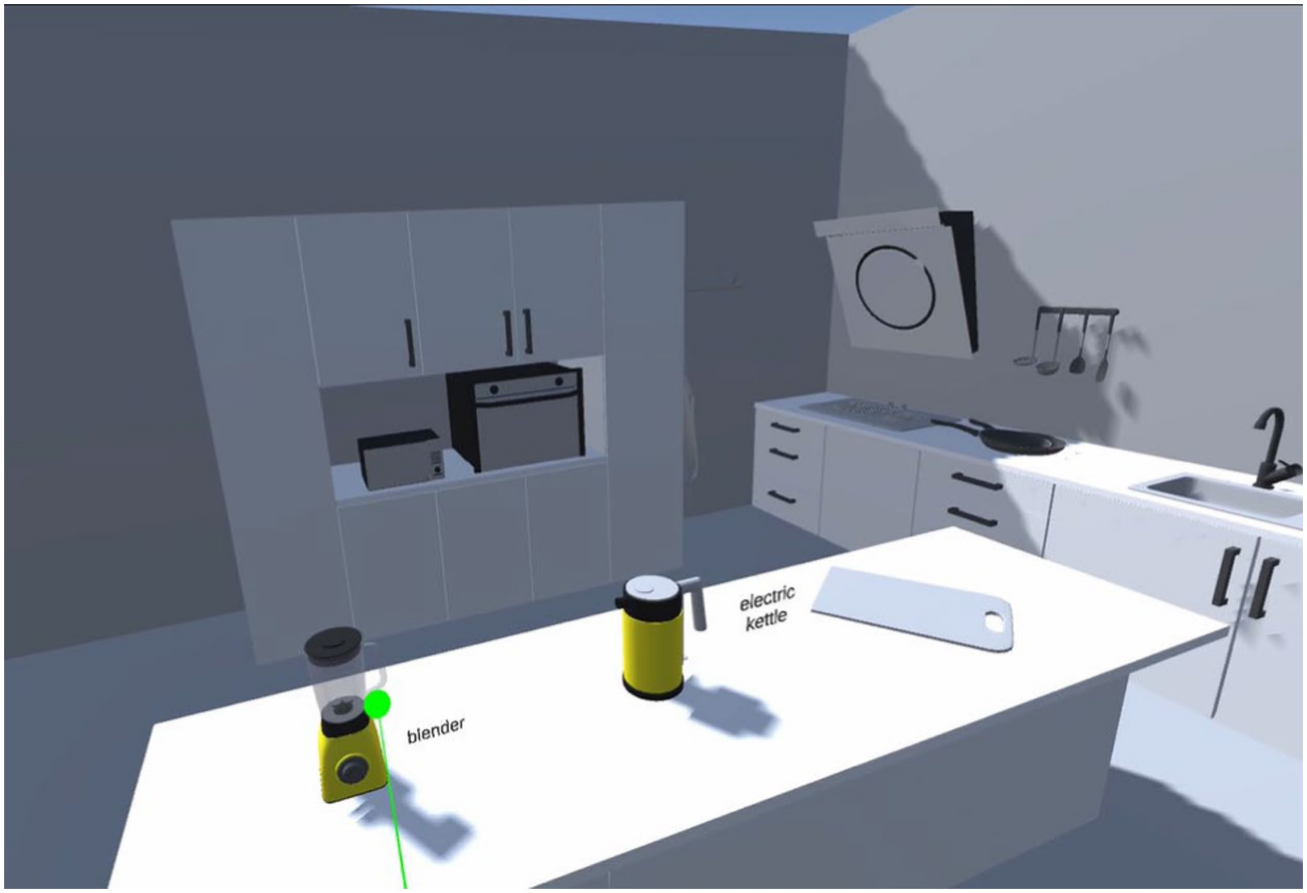
A screen shot of the virtual environment (kitchen).

The learners were first trained in the use of the VR equipment and software. During the intervention, they entered a virtual environment developed by the research team by wearing Helmet-Mounted Displays (HMDs). The virtual scenario was a 150 m^2^ virtual house containing six rooms, including a living room, bedroom, kitchen, bathroom, balcony, and courtyard (Figure 3). 97 interactive virtual objects were displayed in the house (Appendix I). Learners can move their bodies freely in the virtual environment, visit the house and observe each virtual object. Using the controllers, they were able to trigger the virtual objects to bring up a panel containing information regarding the English word corresponding to the object. Meanwhile, learners can hear the pronunciation of the word. Throughout the learning process learners are free to roam in the virtual environment to explore and learn. Any learner who feels uncomfortable is promptly assisted by a research assistant and allowed to exit the experiment at any time.

#### 2.3.2. Control group session

Learners were first trained in the use of the online meeting software. They access the online classroom *via* an app on their PC or smartphone. The instructor can present slides *via* the function of sharing screen. The slides contain target vocabulary, phonetic symbols, Chinese translations, pictures, and sample sentences. The sequence and teaching style of the target vocabulary are designed by the instructor. Some practices were designed, such as word puzzles, where learners can interact with the teacher through their microphones or by responding in the chat room. In addition, the learners can also use the “raise hand” function to get the instructor’s attention, so that they can ask questions. The students in the control group were taught the meaning of the target vocabulary, including their spelling, pronunciation, and usage *via* the conventional approach.

Within 24 h of the end of the intervention for both groups of learners, a post-test with the same tasks and processes as the pre-test was conducted.

### 2.4. Instruments

The development of the VR instructional application used by the experimental group in this study was based on Unity 3D,^1^ a virtual environment development platform. Provision of immersive virtual environment experience for the learners was *via* an HMDs produced by Pico Neo^2^ containing a host displayer, two controllers and a pair of headsets, which also captures the movement of the user’s body, head, and hands to provide a highly immersive experience. The instruction platform used by the control group was DingTalk,^3^ an online conferencing software with the functions of shared screen, whiteboard, and chat room.

### 2.5. Material

#### 2.5.1 Target vocabulary

The target words in this study are related to the theme of furniture and household items (Appendix I). A pilot study had been carried out prior to the pre-test to measure the learners’ mastery of the targeted vocabulary in this study. In ensuring that learners had never been exposed to the vocabulary items used in the treatment, the finalization of including 97 words was based on the outcome of a pilot study, whereby a test was conducted with ten undergraduate students to see their mastery of the targeted vocabulary items. In this pilot study, which involved all 97 target words, students were asked to fill in the blanks with the corresponding Chinese translation of each word and circle the words that they did not know. Based on the pilot study results, 97 target vocabulary words that had not yet been learned were identified as the targeted vocabulary items. Furthermore, the researchers also ensure that the chosen words were not part of the list of terms that the students had learned prior to the study by checking with the students. Additionally, the researchers made sure none of the target vocabulary items is part of the list of words to be learnt based on the course syllabus of their previous English language courses.

#### 2.5.2. Writing task 1

The context of task 1 is that the learners are a tenant who is renting a room. Prior to moving in, they found some unsatisfactory aspects in the rental property, such as missing or damaged furniture or daily appliances. Therefore, they need to produce a list to their landlord, so that these problems will be attended in a timely manner. The materials of the task consisted of 6 photos taken in the house (Appendix II). In case learners cannot see the photos on the answer cards clearly, a QR code was printed on the cards so that they can access the photos by scanning it using their smartphones. These photographs show the living room, kitchen, bedroom, bathroom, and balcony areas of the rental property. To ensure the authenticity of the source of the photographs, they were taken in an actual house to be rented, with the permission of the landlord. As the writing requirements for the task, learners were asked to list as many issues as they noticed in 20 min based on the information provided in the photos. To prevent learners from giving up listing the issues they notice due to vocabulary limitations and thus compromising the completion of the task, these learners were encouraged to write in English, but they are allowed to use their native language when they encounter difficulties (e.g., “There is no 洗衣机 on the 阳台, please buy one.”).

#### 2.5.3. Writing task 2

The context of task 2 is that the learners are a homeowner who is planning to decorate the house. They need to write a letter to the designer, in which they tell him the style of decoration they want and the furniture, decorations and appliances they would like to add. As requirements, learners are expected to include at least the following information in their letters:

- The types and styles of furniture and items to be purchased for each room.
- The furniture and decor they need in each room, including living room, bedroom, bathroom, kitchen, balcony, and garden.

The requirements for language usage are the same as in task 1.

The consideration of setting up the two tasks was to enable learners to demonstrate their vocabulary use and strategy in two different types of writing where possible. Task 1 examines writers’ extraction, description, and summary of information from real-life scenes, while task 2 requires the students to formulate and develop description of furniture as well as explanation in relation to a given prompt. The students have to ensure that their ideas are be supported by reasons, and examples may be drawn from their own experience. The common feature of both tasks is that the context of the writing is related to interior decoration, furniture and household items, and the vocabulary involved is linked to the target vocabulary learned during the intervention. The writing tasks were designed to examine learners’ use of the target vocabulary and their ability to complete the tasks through what they had learned.

#### 2.5.4. Rubric

According to the review article by Dawson (2017), a rubric is usually a framework for grading the quality of student work. In this study, the rubrics of the writing task is a four-level scale (as shown in Appendix III) that evaluates learners’ writing in terms of the 5 dimensions of *Target word usage*, *Lexical density*, *Distribution richness*, *Spelling mistake*, and *Completion of task*. The total score for each writing task was 100, with each of the five dimensions accounting for 20 marks. *Target words usage* refers to the actual number of targeted words used by learners in the essay, which reflects their performance in using the vocabulary learned during the intervention. *Lexical density* was the percentage of furniture, decoration, and appliance-related nouns in the total number of words in the essay. *Distribution richness* relates to the distribution of the target vocabulary used by the learners across the rooms (e.g., if learners used *couch*, *ashtray*, and *apron*, which are distributed in the living room and kitchen, then distribution richness is two). *Spelling mistake* includes words that are misspelled or words that are replaced with Chinese. *Completion of task* is a non-quantitative indicator that is used to measure the extent to which learners complete the objectives of the task. Learners’ essays are marked by experienced English teachers using a rubric and scoring guide that can be found in Appendix III.

#### 2.5.5. Scoring

The writing data is scored by a university English lecturer based on the rubric of the writing task and the results are reviewed by another independent English lecturer to ensure consistency in rating the essays. In general, these scores were decided by both lecturers upon discussion, and in general there is consistency in how both lecturers scored the learners’ writing.

## 3. Results

The means and standard deviations of learners’ writing performance in the experimental and control groups on the pre-test and post-test are presented in Table 2. It can be seen that the two groups of learners scored similarly in the pre-test. After the intervention, the two groups of learners showed different magnitudes of change in their writing performance.

**Table 2:** Descriptive statistics of learners’ scores in the experimental and control groups on the writing test.

	Pre-test				Post-test			
	Task 1		Task 2		Task 1		Task 2	
	*M*	SD	*M*	SD	*M*	SD	*M*	SD
Experimental group	34.17	11.09	30.74	11.20	60.34	14.94	63.90	14.73
Control group	34.19	15.49	33.29	12.09	53.00	11.24	47.78	12.48

### 3.1. Within-group difference

Within-group difference refers to the score variation of the same learner before and after the intervention. A mixed repeated measures ANOVA was conducted to compare scores on the writing task 1 and task 2 at time 1 (pre-test) and time 2 (post-test). The results of multivariate tests on the two writing tasks are shown in Table 3, which demonstrate the effect of time on the vocabulary usage performance and the significance of the interaction between time and treatment. Since the Sig. value in Mauchly’s test of sphericity is less than 0.05, the values of Wilks’ lambda have been checked.

**Table 3:** The results of tests of within-group effects and time*treatment interaction.

Within subjects effect			Value	*F*	Hypothesis df	Error df	Sig.	Partial eta squared
Time	Wilks’ Lambda	Task 1	0.34	53.562c	5	138	0	0.66
		Task 2	0.324	49.678c	5	119	0	0.676
Time*Treatment	Wilks’ Lambda	Task 1	0.804	6.737c	5	138	0	0.196
		Task 2	0.676	11.421c	5	119	0	0.324

**Figure 4:**
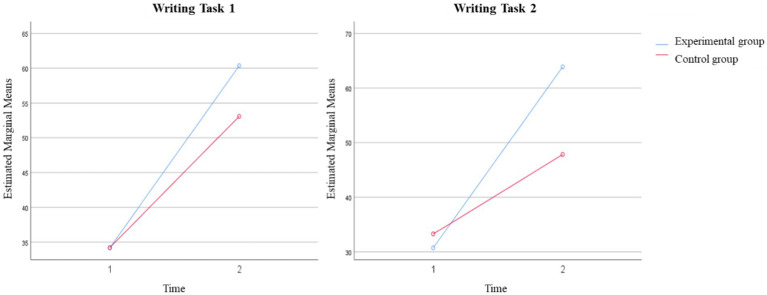
The total writing scores of learners in experimental and control groups at time 1 and time 2.

In writing task 1, the interaction between Time*Treatment was statistically significant [Wilks’ Lambda = 0.804, *F* (5, 138) = 6.737, *p* < 0.001], and the effect for time was moderate [Wilks’ Lambda = 0.34, *F* (5, 138) = 53.562, *p* < 0.001, Partial *η*^2^ = 0.66]. Similarly, in Writing Task 2, the interaction between Time*Treatment was also statistically significant [Wilks’ Lambda = 0.676, *F* (5, 119) = 11.421, *p* < 0.001], and the effect for time was moderate [Wilks’ Lambda = 0.324, *F* (5, 119) = 49.678, *p* < 0.001, Partial *η*^2^ = 0.68]. It suggests that, in Writing Task 1 and Task 2, there were a change in vocabulary usage performance across the two different time periods, whilst the main effect for time was significant. The changes in the total writing scores of the learners in experimental and control groups at two-time levels are shown in Figure 4.

Figure 4 illustrates that the experimental and control groups of learners exhibited varying degrees of improvement in their total scores for Writing Tasks 1 and 2. For both Writing Task 1 and Task 2, according to Figure 4, it is apparent that both the experimental and control groups made significant improvements in their total writing scores, however, the improvement was greater for the experimental group, particularly on task 2. The results are also supported by the manner of vocabulary usage as exhibited by students in the experimental group.

**Figure 5:**
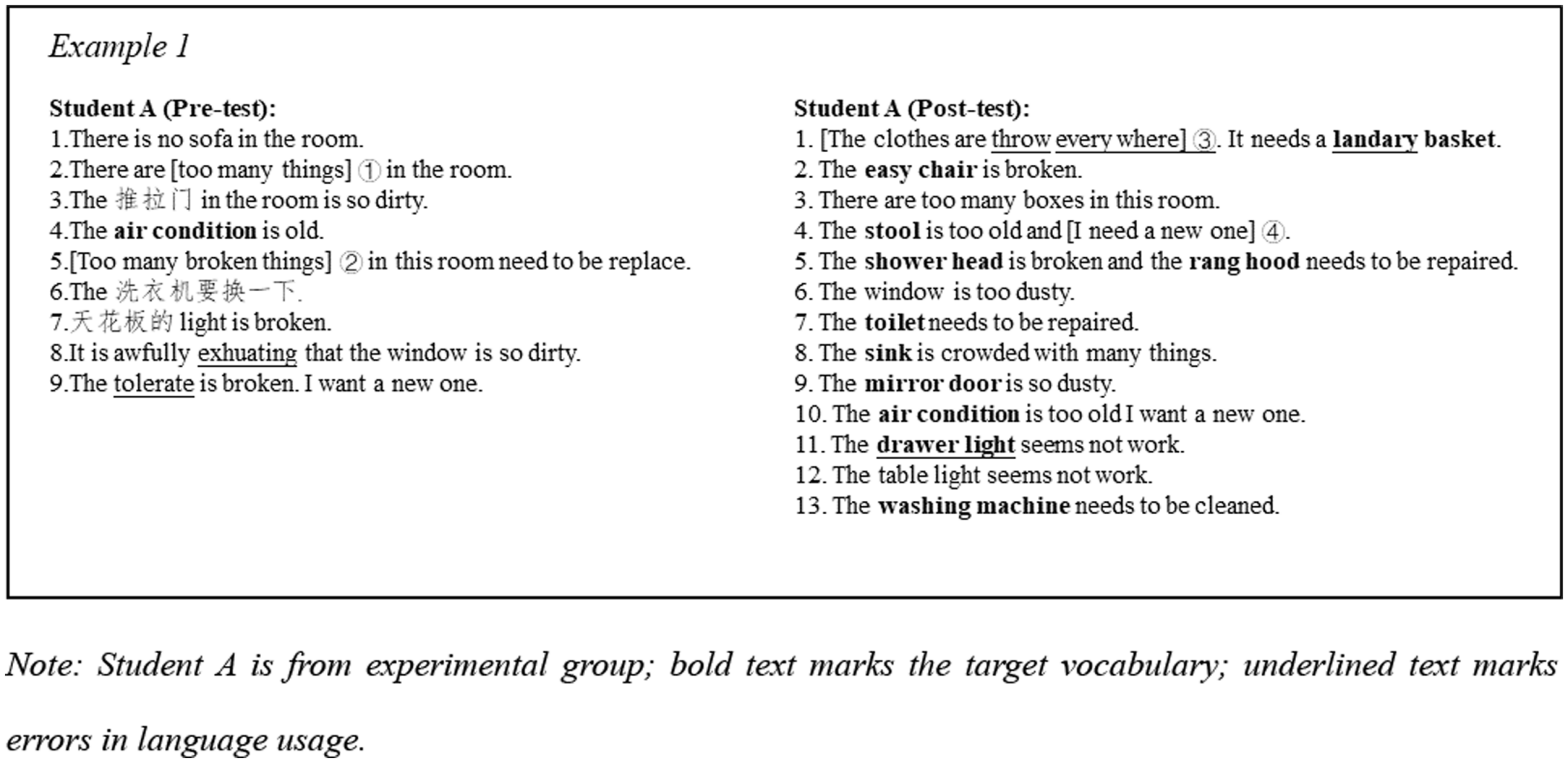
Example 1.

Example 1 (Figure 5) demonstrates the progress of student A’s writing performance in Writing Task 1. The first improvement can be seen at the vocabulary level. At first glance, there is an increase in the number of furniture related words. There are 11 furniture-related words were used in post-test, whereas only 2 in the pre-test. Additionally, 3 Chinese translations were found in the pre-test, which indicated that she did not know how to name some of the furniture when describing the problem. In dealing with her lack of lexical knowledge, student A used “too many things” (shown as mark ①) to express her dissatisfaction with the clutter in her room; similarly, she uses “too many broken things” (shown as mark ②) to describe the damage to some furniture and appliances. This way of describing was first considered as a communicative strategy to compensate for a lack of vocabulary. According to the write up in the pre-test, these learners were found to be unable to provide clear description regarding the pieces of furniture and only managed to describe vaguely the physical aspects of the rooms. However, 11 related words were used in the post-test, while no Chinese was used in the post-test. It suggests that student A has made progress in target words usage and no need to relies on her L1. In terms of completing the task, student A was able to clearly state the requirements in the post-test and give reasons for them. For example, “the clothes are threw everywhere” was the reason for “I need a laundry basket” (shown as mark ③). This makes the presentation of the claim more reasonable and convincing and helps to achieve the task objective. Overall, student A showed improvement in the number of target words used, the ability to describe the problem clearly and the ability to make explicit claims in the post-test compared to her writing in the pre-test. These made the communication more accurate, convincing, and acceptable.

**Figure 6:**
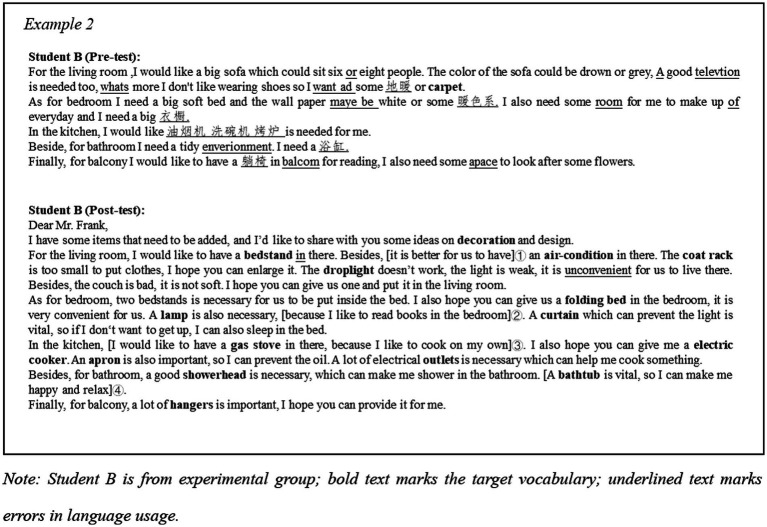
Example 2.

According to Example 2 (Figure 6), which shows student B’s writing performance in Task 2, the improvement can be seen from the vocabulary usage and content enrichment. Eight Chinese words were used in pre-test, such as “浴缸 (bathtub)” and “烤炉 (oven),” which indicates that vocabulary hindered her expressions of idea. However, in the post-test, the same student was able to include many relevant words to make the expressions more accurate. The writing in the post-test is fully in English and 16 target words were used. From the aspect of content, more proposals were made explicit (shown in ①), and also more expressions explaining the reasons. For example, when mentioning the need for a lamp, student B added “because I like to read books in the bedroom” (shown in ②). Another example was given by emphasizing the importance of installing a bathtub, she mentioned that it would make her feel “happy and relaxed” (shown in ④). Ultimately, student B achieved better performance in completing the task.

**Figure 7:**
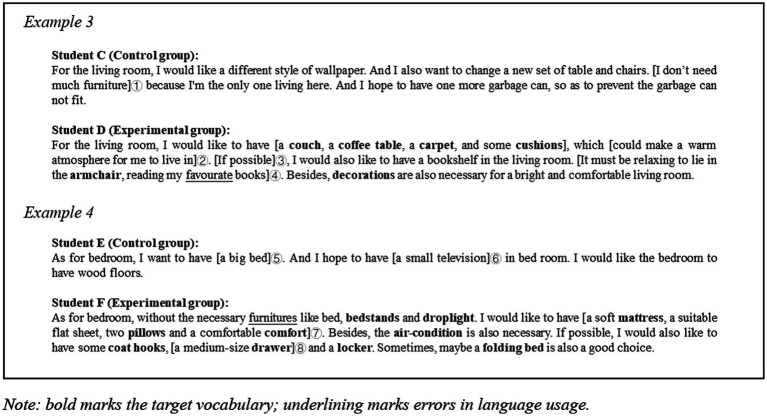
Example 3 and 4.

### 3.2. Between-group difference

**Figure 8:**
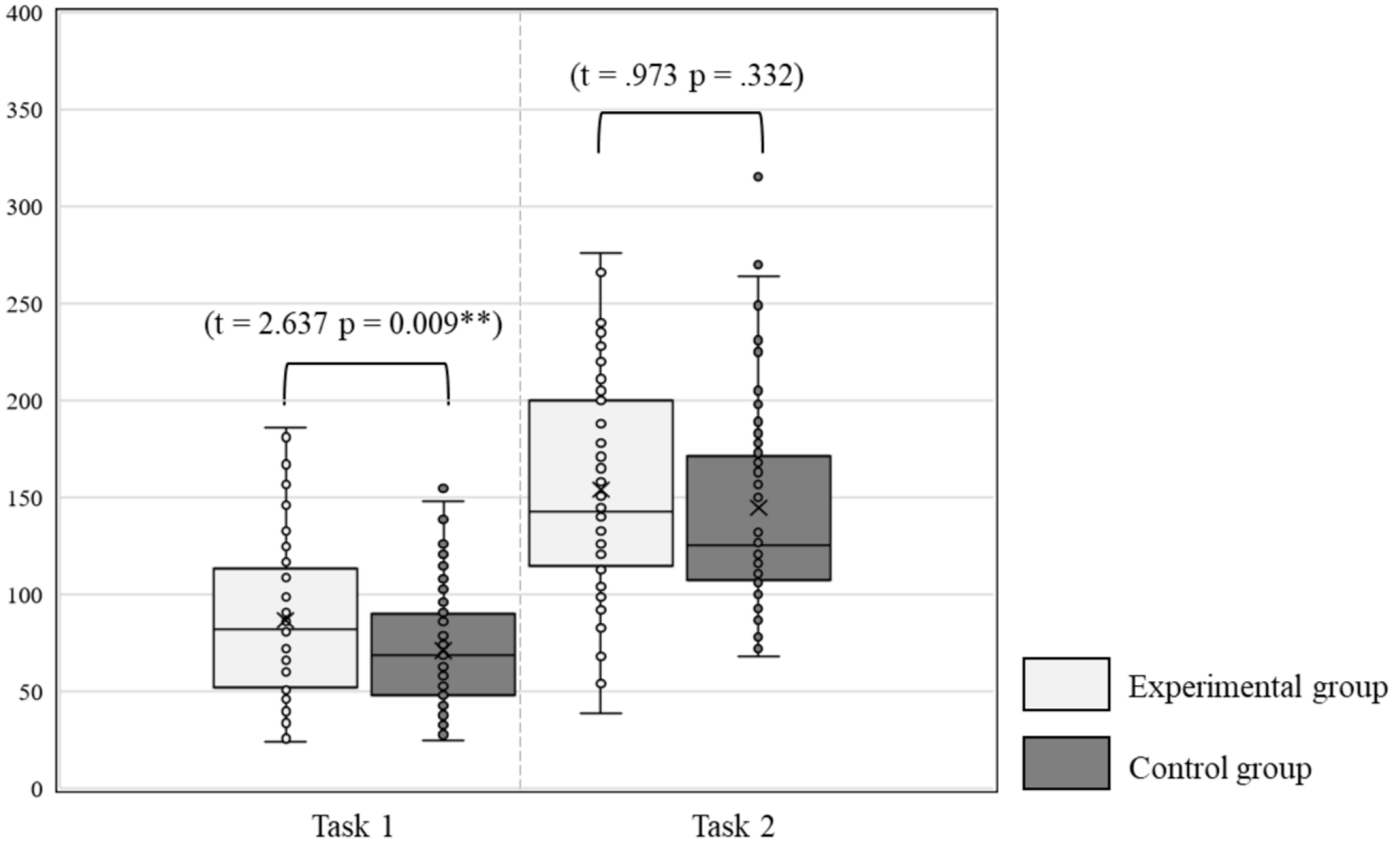
Word counts of experimental and control groups in writing task 1 and 2.

The results showed (as in Figure 8) that in Writing Task 1, learners in the experimental and control groups had 86.77 ± 40.65 and 71.23 ± 29.59 words used in their compositions respectively; learners using VR completed Task 1 with 15.54 more words in vocabulary use (95%CI: 3.89 ~ 27.19) than those in the control group, a statistically significant difference (*t* = 2.637, *p* < 0.01). In contrast, in Writing Task 2, the two groups of learners had similar word counts (Experimental: 154.02 ± 53.60; Control: 144.73 ± 53.00) and the difference in their vocabulary counts was not statistically significant (*t* = 0.79, *p* > 0.05). At first glance, it appears that learners using VR technology used more vocabulary in the writing task requiring extracting, describing, and summarizing from existing information; whereas this difference was not significant when completing writing task based on the imagination. It suggests that learners using VR technology had significantly more length in completing Task 1, whilst this difference was not significant in Task 2.

Furthermore, the differences in learners’ vocabulary usage and writing performance were analyzed based on a mixed repeated measures ANOVA. The Shapiro–Wilk test suggested that the data from both groups demonstrate normal distribution. Table 4 illustrates the differences between learners in the experimental and control groups in writing tasks 1 and 2 after the intervention, including *Target word usage*, *Lexical density*, *Distribution richness*, *Spelling mistake*, and *Completion of task*. In Writing task 1, there was a statistically significant difference one total writing performance between experimental and control groups [*F* (1, 142) = 5.079, *p* = 0.026], and the effect size was small (Partial *η*^2^ = 0.035). Specifically, the two groups of learners differed in Target word usage [*F* (1, 142) = 18.641, *p* < 0.05, Partial *η*^2^ = 0.116]. Besides, their difference in *Distribution richness* was also significant [*F* (1. 142) = 4.881, *p* = 0.029], but the effect size was small (Partial *η*^2^ = 0.033). Notably, the difference between the two groups of learners on *Completion of task* was statistically significant [*F* (1, 142) = 8.665, *p* = 0.004], and the value of Partial *η*^2^ is 0.058. There were no significant between-group differences found in *Lexical density* and *Spelling mistake* (*p* > 0.05). Consequently, for Writing Task 1, there was a significant difference between the two groups in terms of *Target word usage*, but the between-group difference in *Distribution richness* and *Completion of task* were considered small.

**Table 4:** The results of between-group effect (Treatment) within the Mixed ANOVA in writing task 1 and 2.

Transformed variable: average													
		Writing Task 1						Writing Task 2					
Source	Measure	Type III sum of squares	df	Mean square	*F*	Sig.	Partial eta squared	Type III sum of squares	df	Mean square	*F*	Sig.	Partial eta squared
Intercept	Target word usage	3770.532	1	3770.532	436.808	0	0.755	8226.277	1	8226.277	305.463	0	0.713
	Lexical density	38436.038	1	38436.038	1431.194	0	0.91	20818.048	1	20818.048	1281.237	0	0.912
	Distribution richness	22110.997	1	22110.997	718.043	0	0.835	29782.82	1	29782.82	1453.005	0	0.922
	Spelling mistake	53506.311	1	53506.311	2318.795	0	0.942	39513.295	1	39513.295	1512.624	0	0.925
	Completion of task	17737.408	1	17737.408	922.322	0	0.867	7572.967	1	7572.967	1517.215	0	0.925
	Total	593900.831	1	593900.831	3201.115	0	0.958	480751.89	1	480751.89	3127.074	0	0.962
Treatment	Target word usage	160.907	1	160.907	18.641	0	0.116	752.565	1	752.565	27.945	0	0.185
	Lexical density	50.122	1	50.122	1.866	0.174	0.013	180.832	1	180.832	11.129	0.001	0.083
	Distribution richness	150.303	1	150.303	4.881	0.029	0.033	730.596	1	730.596	35.643	0	0.225
	Spelling mistake	0.006	1	0.006	0	0.987	0	185.551	1	185.551	7.103	0.009	0.055
	Completion of task	166.63	1	166.63	8.665	0.004	0.058	0.999	1	0.999	0.2	0.655	0.002
	Total	942.331	1	942.331	5.079	0.026	0.035	2839.698	1	2839.698	18.471	0	0.131
Error	Target word usage	1225.746	142	8.632				3312.459	123	26.931			
	Lexical density	3813.541	142	26.856				1998.552	123	16.248			
	Distribution richness	4372.666	142	30.793				2521.18	123	20.497			
	Spelling mistake	3276.657	142	23.075				3213.049	123	26.122			
	Completion of task	2730.838	142	19.231				613.937	123	4.991			
	Total	26345.169	142	185.529				18909.846	123	153.739			

In Writing Task 2, it was found that learners using VR had significantly higher total writing scores than learners in conventional instruction [*F* (1, 123) = 18.471, *p* < 0.05], and the effect size is large (Partial *η*^2^ = 0.131). Align with Task 1, the two groups of learners were found to have marked differences in Target word usage [*F* (1, 123) = 27.945, *p* < 0.05, Partial *η*^2^ = 0.185]. However, unlike the results for Task 1, the difference was statistically significant in Lexical density, Distribution richness, and Spelling mistake for both groups of learners. According to their Partial *η*^2^ values, the subject effect size for Distribution richness (0.225) was very large and for Lexical density (0.083) and Spelling mistake were moderate (0.055). Notably, both groups’ learners did not differ significantly on completion of task. In short, the results demonstrate that there was a significant difference in the total scores between the experimental and control groups. It suggests that the between-group difference was small in Task 1 and large in Task 2. In terms of content produced by learners, there is an evident difference in terms of vocabulary richness.

Examples 3 and 4 (Figure 7) demonstrate the fragments of writing about the same room for both groups of learners in Task 2. They show the differences in vocabulary richness and strategies used by the two groups of learners in describing similar scenes. For living room, in Example 3, student D from the experimental group had specific demands for a *couch*, *coffee table*, *carpet* and *cushions* to reflect a warm atmosphere (as shown in ②); in contrast, student C in the control group expressed a desire for “a new set of tables and chairs.” Although they both may have some degree of similarity in their intrinsic needs, student D is more detailed and precise in his expression. This difference seems to be even more evident in Example 4. When referring to items that needed to be added to the bedroom, student E from the control group suggested “a big bed” and “a small television” (as shown in ⑤ and ⑥). Presumably, the sentence expresses her concern about the bed and television in the bedroom and her desire to draw the designer’s attention to them. However, the use of the adjectives *big* and *small* here makes the statement fuzzy. Perhaps student E’s actual needs were for a comfortable bed and a wall-mounted television that would fit in the bedroom, but as a result she drew the reader’s attention to the size. As comparison material, similar claims were found in the experimental group’s writing data. For example, student F also made a need for a bed when she wrote “… a soft mattress, a suitable flat sheet, two pillows and a comfortable comfort.” (shown in ⑦). Student F enriched her specific request for a bed with a clear statement.

Similar details are also available from “a medium-sized drawer” (shown in ⑧). At the discourse aspect, student C was found to use “And” frequently as an articulation word, which is not in line with the discourse norms of letter writing, while student D used “if possible” (as shown in ③) and “besides.” Student D shows more engagement with communication, which refers to the fact that she seems to be communicating her concerns and ideas with a real-life reader. She made more references to herself, including her feelings, hobbies (as shown in ④), enabling the designer to be more aware and receptive to her requests. The addition of this information contributes to a better fulfillment of the task objectives.

## 4. Discussion

**Figure 9:**
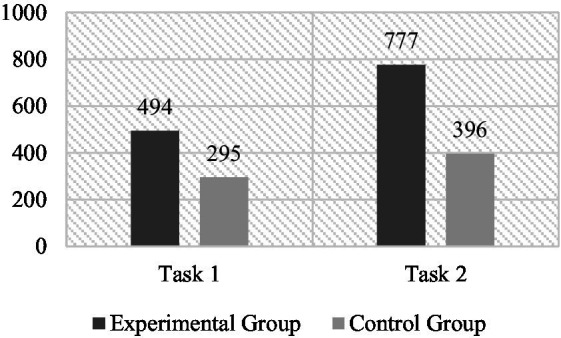
The number of the target words used in writing task 1 and 2 between experimental and control groups.

In second language instruction, the purpose of vocabulary teaching is not only to efficiently memorize the meaning and spelling of target words, but also to enable learners to use them appropriately in real word applications. EFL learners have difficulty in achieving the vocabulary breadth and quality of native speakers in practice (Hulstijn, 2001), and have been found to rely more on 1^st^ 1,000 words of the Lexical Frequency Profile (LFP) when completing writing tasks (Zhai, 2016). In this study, learners using IVR were found to achieve better performance in vocabulary usage in their writing meant for actual application, as evidenced using more target words (see Figure 9) and a higher lexical density in writing tasks. These findings are in line with Lan et al. (2019) results. The reasons for this result may be multifaceted. Learners who used IVR may have acquired better short-term memory, which means that they could remember more words upon completing the VR sessions (Tai et al., 2020). This may cause more confident in experimental group learners in using these newly learned words. In addition, more informative writing may be another important reason for the increased use of target vocabulary. As the comparison between Examples 3 and 4 (Figure 7), the writings from the experimental group have more details being mentioned. This has indirectly led to an increase in the number of target words. Moreover, learners using the IVR were found to use target vocabulary that are located in more rooms. Learners who scored over 16 points described design ideas for at least four rooms to the designer. It is another aspect that demonstrates the openness of the ideas of the experimental group. In Example 2 (Figure 6), the 15 target words student B used were located in the living room, bedroom, kitchen, and bathroom. In contrast, the words used by learners in the control group are quite limited, i.e., they are confined to furniture located in 2 rooms on average according to the obtained data. The higher *Distribution richness* obtained by the experimental group may be related to their sense of presence in the virtual environment. Learners were allowed to move and explore freely in the six virtual rooms, resulting in an impressive experience of embodiment. While completing the real-world task, learners were able to retrieve words by recalling their experiences of exploration, which were the sights they saw when they were in the six rooms. Meanwhile, the experience of moving between the rooms brings them an overall sense of the house. This provided the basis for learners to retrieve words from different scenes. Furthermore, this spatial memory may also facilitate learners’ creativity and discourse construction in the task, especially when describing their ideas for decoration to interior designers.

Learners using IVR techniques achieved significantly higher scores of *Completion of task*. It is closely related to the accuracy with which learners describe problems and express ideas, which is directly related to task completion. In line with Huang et al.’s (2019) research findings, learners using IVR generally demonstrated better communication intentions and strategies, as well as more creative tendency. The experimental group’s learners were found to present more detail and were able to present their ideas and claims clearly. This certainly contributes to the task, as the interior designer can get a clearer picture of the writer’s needs from the reading. In contrast, learners in the control group tended to use more vague expressions such as “I want a new table” or “I would like a big bed.” These expressions were not conducive to task completion because the information it conveys is ambiguous. In addition, learners in the experimental group were found to be more willing to explain the reasons for their requests and to refer to their own preferences and feelings more frequently. For example, “I would like to install a bookshelf because I like reading” or “I need a bathtub so that I can relax myself.” These expressions made their ideas and requests easier to be understood and accepted by the interior designer, who is the imaginary reader in this task. This finding is consistent with the findings of Chen et al. (2020) study, whereby more description, causes, and enumeration were found in their writing. Overall, learners who had VR experiences achieved significantly better writing performance. However, the findings of this study differ from that of Dolgunsöz et al. (2018) who concluded that VR-based learning experiences had no effect on short-term writing performance. Their study showed that learners’ scores on the writing tasks in the immediate-posttest did not differ significantly from those in the pre-test, which also corresponds with the findings of another study by Huang et al. (2019).

Considering that participants in this study were all first-year university students who lived in accommodation on campus, they have few experience in dealing with matters related to the renting and interior design, and researchers like Yang et al. (2021) also found that learners’ inner motivation and interest in writing are impacted by the lack of experience in dealing with matters related to topics of writing. As such, it will not be surprising to find students use generalizations and did not seem to know much about the furniture items when talking about a topic that is not often touched upon. Nevertheless, learners in the experimental group still obtained better performance, which may be related to the learning experience in the virtual environment. An experience related to the subject of the writing is considered to have a crucial role to play in enhancing the performance of the writing. The writers are more likely to provide vivid details and his or her feelings when expressing a prior experience. This certainly contributes to the richness of the writing and makes it more compelling. Conversely, when writers base their expressions solely on their knowledge, the expressions can appear stiff and hollow. In EFL instruction, teachers are keen to create specific contexts and themes to support teaching and learning in order to provide learners with the appropriate context. However, it is crucial to get learners to believe in and engage with the context set by the teacher, which is the difficulty of the instruction. Based on the CAMIL theory (Makransky and Petersen, 2021), IVR technology can facilitate contextual teaching by providing a high level of immersion, thus transforming different forms of knowledge into an experience. The virtual environment constructed by the IVR technology gives the learner a sense of ‘being there’ and an embodied experience of exploring the environment, a process that allows the learner to form a memory about the event, which contains a wealth of information between word, space, and learners’ bodily experience. In contrast, conventional classroom instruction focuses almost exclusively on the target vocabulary knowledge, and does not offer any of the premonition aspects. Although some teaching methods may be used to promote learning, they cannot be transferred into the learner’s experience. In this study, embodied experience was found to influence short-term retention of vocabulary memorizing. The IVR technology simulated a scenario to enable learners to have an exploratory experience in which knowledge and context were revealed. In contrast to memorizing abstract knowledge, learners using VR technology were able to retrieve knowledge by recalling a pathway through their own exploratory experience. In completing writing tasks, these experiences are the scaffolding for learners’ creativity and expression. Consequently, learners are better able to utilize relevant vocabulary and spatial knowledge to construct the content of their current writing. As a result, this experience facilitated the transfer of vocabulary learning. They were able to apply what they had learned in solving real-world problems, resulting in better writing performance. The above pathways demonstrate how the process improves learners’ vocabulary usage and writing.

Certain aspects of VR may adversely impact the manner some participants’ experience. The participation in VR environment impacted the physical well-being of some learners. For instance, some participants experienced mild vertigo during the use of VR, which directly interfered with their process of learning. While other participants mentioned that they were unable to jot down notes in the virtual environment, which caused problems for the retention of the words learnt. Notably, although learners in the experimental group used more of the target vocabulary, they made significantly more spelling errors than the control group. One possible cause for this is that learners in the conventional classroom had ample time to memorize the spelling of a vocabulary word and take notes, whereas learners exploring the virtual environment cannot focus too much on the spelling of the word. The lack of teacher guidance and differences in memory patterns could be other potential factors. Exploring this issue in future research is warranted as it will help to understand whether VR-based learning enhances accuracy of knowledge retention. Additionally, future studies may also want to consider setting up multiple experimental groups using VR devices with different levels of immersion, as well as assessing performance across multiple time lines. Future researcher may also wish to includes interview sessions to find out which particular aspects of IVR notably influence the learning process.

## 5. Conclusion

The implication of this study for EFL instruction is that VR-assisted language learning facilitates the transfer of knowledge into practical language usage. With IVR technology, learners generate a sense of immersion in the virtual environment and gain an experience of embodiment. Therefore, virtual reality can be considered for teaching and learning that promotes EFL learners’ output skills, such as speaking and writing. However, the instructor should also be concerned about the potential for reduced memory accuracy, such as more spelling mistakes. In practice, teachers need to be aware of the individual differences in learners. VR-assisted language learning is not suitable for learners who are dependent on teachers and peers, or for learners who are anxious or unable to concentrate adequately in the virtual environment due to the novelty or lack of knowhow dealing with VR related learning process. The addition of supplementary instruction processes is worth considering, such as guiding learners to review what they have learnt in VR after the class. Although there are important discoveries revealed by these studies, there are also limitations. Considering that all participants were from the same university, this left the sample underdiverse. Additionally, the novelty effect needed to be considered, since most of the participants used the IVR devices for the first time.

This study compared the vocabulary usage and writing performance of learners using IVR technology and in a conventional classroom. The improvement was found in both groups, but the differences between groups were statistically evident. These differences could be linked to learners’ experiences in the virtual environment. This is related to the sense of presence, i.e., the learners believe they are part of the virtual space and that affects their learning performance. Based on the sense of presence, it triggers the learners’ sense of embodiment that helps to explain the process whereby learners connect themselves to entities in the virtual environment. This altered psychological state (e.g., a dream or hallucination, Riva et al., 2003) enabled learners to transform their learning process into an embodied experience. Evidently, what causes the improvement in writing performance is due to the learners’ virtual experience and their memory of the event. Therefore, future studies may want to investigate the potential of embodiment experience to enhance learning of different subject matters.

## Data availability statement

The raw data supporting the conclusions of this article will be made available by the authors, without undue reservation.

## Ethics statement

The studies involving human participants were reviewed and approved by Universiti Malaya Research Ethics Committee, Universiti Malaya. The patients/participants provided their written informed consent to participate in this study.

## Author contributions

Under the guidance of LN, BF has completed the study design. BF worked independently on the preparation of the experimental materials and the data collection phase, as well as the quantitative analysis of the experimental data. LN and BF worked together on the qualitative analysis and the writing of the manuscript. LN proofread the findings and the manuscript. All authors contributed to the article and approved the submitted version.

## Conflict of interest

The authors declare that the research was conducted in the absence of any commercial or financial relationships that could be construed as a potential conflict of interest.

## Publisher’s note

All claims expressed in this article are solely those of the authors and do not necessarily represent those of their affiliated organizations, or those of the publisher, the editors and the reviewers. Any product that may be evaluated in this article, or claim that may be made by its manufacturer, is not guaranteed or endorsed by the publisher.

## Footnotes

^1^ https://unity.com

^2^ https://www.pico-interactive.com

^3^ https://www.dingtalk.com
