# Supplementary material for: Facilitating writing performance of EFL learners via virtual reality: Immersion, presence, embodiment
Source: Front Psychol. 2023 May 5;14:1134242. doi: 10.3389/fpsyg.2023.1134242 (PMC10197927; doi:10.3389/fpsyg.2023.1134242)
Supplement: Supplementary file 1 [file Data_Sheet_1.pdf]

Appendix I *Target Vocabulary List*

balcony	stool	toilet	hanger	pan
cupboard	bench	urinal	threshold	ladle
faucet	easy chair	electric kettle	mattress	blender
bathtub	folding bed	electrical outlet	louver	broom
washing machine	spring	heating	curtain	dustpan
refrigerator	bedspread	tile	couch	mop
microwave oven	comforter	timber	ashtray	range hood
electric cooker	pillow	shovel	vacuum	hanging cabinet
air-condition	ladder	balustrade	razor	sink
switch	chopping board	decoration	hair dryer	clothespin
ceiling	worktop	ottoman	hairbrush	apron
brick	oven	stacking chair	toothpaste	coffee table
marble	gas stove	headboard	cotton swab	watering can
pipes	screw	footboard	nail clipper	cushion
drawer handle	hat rack	bedstead	laundry basket	parquet
towel rack	mirror door	droplight	towel bar	gravel
coat hook	shoe rack	ceiling light	paper holder	bulb
carpet	drawer	paint	trash can	mirror light
lockers	bedstand	showerhead	armchair	floor lamp
washbowl				

Appendix II *Writing Task 1 & 2*

Welcome to participate in this writing task!

Task 1 （20 min）

Imagine that after graduation, you study abroad and rent a house in your dream city. It will be your living space. The following 6 photos were taken in your rented house. You will find that some equipment is damaged and some equipment is missing, such as furniture, electrical appliances, and daily necessities. These problems will affect your living experience! Therefore, please make a list for your landlord, Mr. Johnny, and tell him the current housing problems and what necessary facilities he needs to add to you.

note:

Please list as many problems as possible in the house so that your landlord can improve before you move in. You will be encouraged to write in English, and you can substitute your native language when you encounter difficulties. *(e.g., There is no 洗衣机 on the 阳台, please buy one.)*

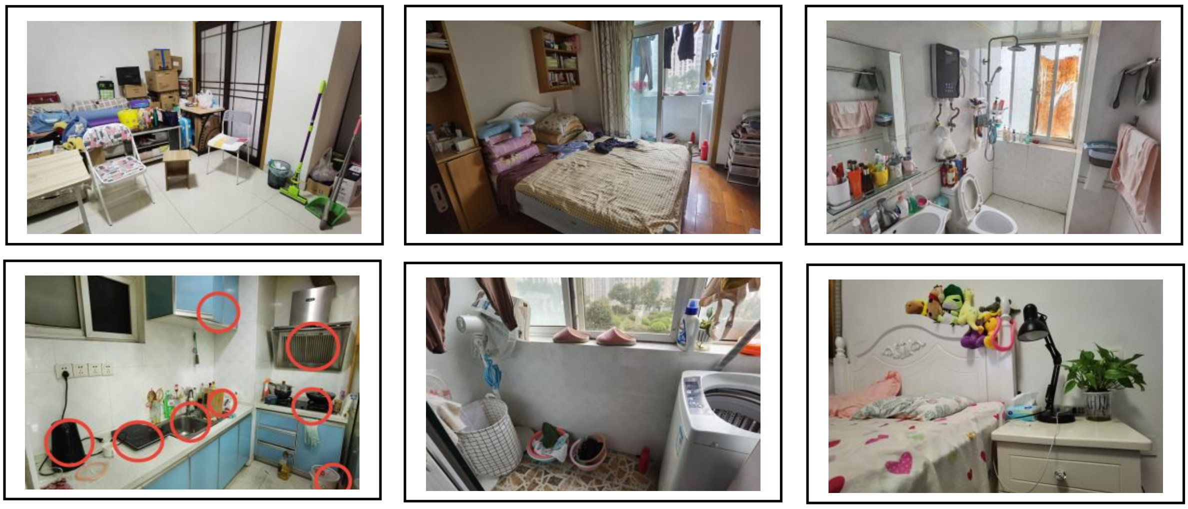

Task 2 (40 min)

Congratulations on successfully completing the previous task! After you communicate with the landlord, your house has been repaired and improved. Now, you plan to start decorating your room to make it warm and comfortable. On the issue of decorating and procurement, you need to communicate with your designer friend Mr. Frank. You need to tell him:

1. The types and styles of furniture and items to be purchased for each room.
2. The furniture and decor you need in each room, including living room; bedroom; bathroom; kitchen; balcony and garden.

note:

You will be encouraged to write in English, and you can substitute your native language when you encounter difficulties. The picture below is a design drawing of your house, which may help you.

Appendix III *The rubric of the writing task.*

CATEGORY	16-20	11-15	6-10	1-5
**Target words usage**	There are more than 16 target words in *appendix I* used in the writing task.	There are 11-15 target words in *appendix I* used in the writing task.	There are 6-10 target words in *appendix I* used in the writing task.	There are 1-5 target words in *appendix I* used in the writing task.
**Lexical density**	Vocabulary related to furniture and interior design accounted for more than 15% of the total number of words.	Vocabulary related to furniture and interior design accounts for 10%-15% of the total number of words.	Vocabulary related to furniture and interior design accounts for 5%-10% of the total number of words.	Vocabulary related to furniture and interior design accounted for less than 5% of the total number of words.
**Distribution richness**	The target words used in the writing are distributed in more than 4 rooms of the virtual environment.	The target words used in the writing are distributed in 3 rooms of the virtual environment.	The target words used in the writing are distributed in 2 rooms of the virtual environment.	The target words used in the writing are distributed in only 1 room of the virtual environment.
**Spelling mistakes**	There are less than 3 vocabulary spelling mistakes in the writing task.	There are 3-5 vocabulary spelling mistakes in the writing task.	There are 6-8 vocabulary spelling mistakes in the writing task.	There are more than 8 vocabulary spelling mistakes in the writing task.
**Completion of Task**	Writer describe the issues or expresses an idea with a high degree of accuracy; communicates effectively with the recipient; demonstrates a positive intent to complete the contextual task; the content of the writing contributes to the solution of the problem and clearly presents their solution. Demonstrates a creative perspective in the course of the task.	Writer describe the issues or expresses ideas with a high degree of accuracy and communicates effectively with the recipient; demonstrates a positive intent to complete situational tasks; most of the writing contributes to problem solving.	Writers are less accurate in describe the issues or expressing ideas; a sense of communication with the recipient exists but is less effective; lightly demonstrates an intention to complete situational tasks; a small proportion of the writing contributes to problem solving.	The writer is barely able to describe the issues or express ideas accurately; he/she does not communicate effectively with the recipient; he/she does not demonstrate an intention to complete the contextual task; and the content of the writing does not contribute to the solution of the problem.

Notes:

Target words usage: 1 mark for each targeted word used, up to a maximum of 20 marks.

Lexical density: Ratios between 1% and 20% (approximate) correspond to 1 to 20 points respectively. Those exceeding 20% are awarded 20 points.

Distribution richness: The number of rooms distributed has 6 possibilities: 0, 1, 2, 3, 4, 4+ (more than 4), corresponding to 0-20 of the rubrics. Thus, 0-20 is divided equally into 5 groups, forming an arithmetic progression with a tolerance of 4. The following table shows how the number of rooms corresponds to the number of points：

Number of rooms	0	1	2	3	4	4+
Marks	0	4	8	12	16	20

Spelling mistakes: 20 marks for no spelling errors; minus 2 marks for each additional spelling error; 0 marks for more than 10 spelling errors.

Completion of task: Scored based on rubric’s description of the standards in different grades.
